# C1QTNF6 promotes oral squamous cell carcinoma by enhancing proliferation and inhibiting apoptosis

**DOI:** 10.1186/s12935-021-02377-x

**Published:** 2021-12-14

**Authors:** Xiaobin Song, Longjie Li, Liang Shi, Xinyu Liu, Xun Qu, Fengcai Wei, Ketao Wang

**Affiliations:** 1 Department of Oral and Maxillofacial Surgery, Qilu Hospital of Shandong University, Jinan, 250012 China; 2 Institute of Stomatology, Shandong University, Jinan, 250012 China; 3 Department of Oral Medicine, Qilu Hospital of Shandong University, Jinan, 250012 China; 4 Institute of Basic Medical Sciences, Qilu Hospital of Shandong University, Jinan, 250012 China

**Keywords:** Oral squamous cell carcinoma (OSCC), C1QTNF6, Acute Phase Response signaling pathway

## Abstract

**Background:**

C1QTNF6 (CTRP6), a member of the CTRP family, has recently been implied to play a role in the tumorigenesis of for a variety of cancer types. However, the role of C1QTNF6 in oral squamous cell carcinoma (OSCC) and its potential molecular remains unclear.

**Methods:**

C1QTNF6 expression was detected by qRT-PCR and western blot analysis. Lentiviral vectors were constructed to knockdown C1QTNF6 in CaL27 and SCC-9 human OSCC cell lines. Cell viability, cell cycle and cell apoptosis analyses were performed by MTT assay, PI/Annexin V staining, and flow cytometry. The effect of C1QTNF6 knockdown on in vivo tumorigenicity of OSCC cells in vivo was evaluated using nude mouse xenograft tumor model. Downstream signaling mechanisms were identified by microarray and Ingenuity Pathway Analysis.

**Results:**

Immunohistochemistry of OSCC tissue and data from TCGA demonstrate that C1QTNF6 was overexpressed in OSCC tissues, and that cellular proliferation was significantly decreased after C1QTNF6 was knockdown in CaL27 and SCC-9 cell lines. Knockdown of C1QTNF6 also resulted in cell cycle arrest at the G2/M phase and enhanced cell apoptosis in in CaL27 and SCC-9 cell lines. Furthermore, knockdown of C1QTNF6 in Cal-27 cells inhibited tumor growth of OSCC in vivo. Microarray analysis revealed that C1QTNF6 silencing resulted in significant alterations of gene expression, with the Acute Phase Response signaling pathway significantly activated following C1QTNF6 silencing.

**Conclusions:**

These results suggest that C1QTNF6 plays an important role in promoting OSCC tumorigenesis, which indicates that C1QTNF6 may comprise a promising therapeutic target for OSCC treatment.

**Supplementary Information:**

The online version contains supplementary material available at 10.1186/s12935-021-02377-x.

## Background

Oral squamous cell carcinoma (OSCC) is the most common epithelial malignancy in the oral cavity, accounting for almost 90% of oral malignancies [1]. Despite improvements in surgical and other therapeutic strategies, the 5-year survival rate of OSCC patients is still disappointing [2, 3]. Thus far, limited information is available regarding the contributing factors and molecular pathways in OSCC tumor formation, and the molecular events have not been defined precisely. Therefore, a better understanding of the molecular carcinogenesis of OSCC may provide new insights on the development of novel diagnostic and therapeutic strategies to improve OSCC patient prognosis.

The C1q/tumor necrosis factor‑related protein family (CTRPs/C1QTNF), including 16 members, contains four domains: a N-terminal signal peptide, a collagenous-like domain, a short variable region, and a C-terminal globular domain [4]. CTRPs and adiponectin share a common structure consisting of a signal peptide at the N terminus, a short variable region, a collagenous domain, and a C-terminal globular domain that is homologous to complement component 1q [5]. Each CTRP has a unique tissue expression profile [11], with C1QTNF6 (CTRP6) widely expressed in many tissues of rodents and primates, including adipose tissue, brain, spleen, lung, liver, muscle, and ovary [6–10]. C1QTNF6 is secreted in a serum-forming oligomeric form [12] and is involved in diverse physiological processes ranging from metabolism to host defense and organogenesis [13]. It has been reported that C1QTNF6 can stimulate fatty acid oxidation via activation of AMPK [14]. In C1QTNF6^−/−^ mice, C1QTNF6 was demonstrated to serve as a novel regulator of the complement alternative pathway with potential to treat rheumatoid arthritis in clinical studies [15]. However, recent studies indicate that C1QTNF6 may have a role in carcinogenesis. One study showed that CTRP4 promotes tumor survival through upregulation of IL-6 and TNF-alpha [16]. Recently, C1QTNF6 was shown to be overexpressed in clear cell renal cell carcinoma, with correlation between elevated C1QTNF6 expression and clinical progression. C1QTNF6 is involved in promoting proliferation and migration as well as in reducing the apoptosis of gastric carcinoma cells and NSCLC [18, 19]. In the latter study, inhibition of C1QTNF6 also was shown to induce G2-M cell cycle arrest [18]. Consistently, C1QTNF6-related signaling pathways activated in ccRCC are enriched in the DNA replication, Cell Cycle, EMT, and angiogenesis signaling pathways [17]. In particular, C1QTNF6 has been shown to be overexpressed in hepatocellular carcinoma tissues and many HCC cell lines, and C1QTNF6 has been shown to promote neovascularization in transplanted HCC cells [20]. This evidence suggests a potential regulatory role of C1QTNF6 in tumorigenesis, which emphasizes the need for further study of the function and mechanism of C1QTNF6 in other cancers.

In this study, we analyzed The Cancer Genome Atlas (TCGA) and found that C1QTNF6 might be a potential tumor-associated regulator involved in the carcinogenesis of OSCC. Furthermore, we confirmed the high expression of C1QTNF6 in OSCC tissues by immunohistochemical analysis and positive expression in OSCC cell lines by RT-PCR. We performed C1QTNF6 knockdown in OSCC cell lines and xenografted tumors to explore the pathological relevance between C1QTNF6 expression and OSCC, including its potential role in the proliferation and apoptosis of OSCC cells in vitro and tumor growth in vivo. We also performed signal pathway analysis to explore the possible molecular mechanisms underlying the regulatory function of C1QTNF6 in OSCC by Ingenuity Pathway Analysis (IPA). The data suggest that the Acute Phase Response signal pathway might play a role in C1QTNF6-mediated promotion of OSCC tumorigenesis.

## Materials and methods

### Cell lines

CAL27 and SCC-9 head and neck squamous cell carcinoma cell lines were cultured in DMEM (Gibco, USA) supplemented with 10% fetal calf serum (Thermo Fisher Scientific, USA), 100 μg/ml streptomycin (Gibco, USA), and 100 U/ml penicillin (Gibco, USA). Human immortalized oral epithelial cell (HioEC) were cultured in a defined keratinocyte-SFM medium (Gibco,USA). Cells were maintained in a fully humidified atmosphere containing 5% CO_2_ at 37 °C.

### TCGA data processing

For expression analysis, we selected RNAseq and RNAseqV2 paired sample data. The raw sequencing data and pathological information were downloaded from TCGA database (https://cancergenome.nih.gov/). For head and neck squamous cell carcinoma (HNSCC), there were 528 samples with available data in the TCGA database, including 520 RNAseqV2 samples, and 40 pairs with sample data and pathological information. Our expression profile analysis was based on these 40 paired samples of RNAseqV2 data. The Trimmed Mean of M-values (TMM) method was applied for data standardization. To avoid errors caused by inappropriate sample grouping, the BCV (biological coefficient of variation) was observed for quality control.

First, we estimated the dispersion of multiple pairs of samples, and then we used a general linear model to estimate whether there was a difference in genes between different groups. Genes with a statistical test P value less than 0.05 were considered differentially expressed genes. The fold changes (FCs) in expression were calculated, and differentially expressed genes with log2|FC|≥ 1.0 (Cancerrmal) were considered to be significant. The remaining genes were filtered out. Additionally, several other criteria were adopted to filter genes: reported functional and clinical relation in HNSCC; multiple transmembrane proteins; those for which gene annotation was not clear (such as the open reading frame); and those reported in more than 100 articles in PubMed. The final gene list was randomly concentrated, and C1QTNF6 was selected for further experimental research.

### Lentivirus construction and lentiviral transfection of CAL27 cells and SCC-9 cells

C1QTNF6-targeting short hairpin RNA (shRNA) oligonucleotide sequences (TGTGTGAGATCCCTATGGT) were designed, synthesized and cloned into the pGV115-GFP vector by GeneChem Corporation (Shanghai, China). Scrambled shRNA (TCTCCGAACGTGTCACGT) was also inserted into the pGV115-GFP vector and used as the negative control. Lentivirus transfection was performed according to the manufacturer’s recommended protocol. CAL27 and SCC-9 cells were seeded into a six-well plate (~ 1 × 10^5^ cells per well) and incubated at 37 °C with 5% CO_2_ until they reached ~ 70% confluence. Then, lentivirus was added at MOI 1. After 72 h, cells were observed under a fluorescence microscope and harvested for subsequent experiments.

### RNA extraction and quantitative real-time PCR (qRT-PCR)

Total RNA was isolated from cells using TRIzol (Invitrogen, USA) according to the manufacturer’s instructions. Reverse transcription was performed to synthesize cDNAs using M-MLV reverse transcriptase (Promega) and OligodT primers (Sangon, Shanghai). C1QTNF6 mRNA expression was detected by quantitative real-time PCR using SYBR Master Mixture (Takara, Japan) on a Real-Time PCR machine TP800 (Takara). The cycling parameters were: 95 °C for a 30 s hot start followed by 45 cycles of 95 °C for 5 s and 60 °C for 30 s. Primers used were as follows: GAPDH forward: 5′-TGACTTCAACAGCGACACCCA-3′, GAPDH reverse: 5′-CACCCTGTTGCTGTAGCCAAA-3′, C1QTNF6 forward: 5′-GAAAGGGTCTTTGTGAACCTTGA-3′, and C1QTNF6 reverse: 5′-CTGCGCGTACAGGATGACAG-3′. The relative mRNA expression was calculated using the 2^−ΔΔCt^ method.

### Western blot analysis

Proteins were extracted, and the protein concentration was adjusted to 2 μg/μL and stored at − 80 °C for later use. Total protein was separated by 10% sodium dodecyl sulfate polyacrylamide gel electrophoresis (SDS-PAGE) and transferred to polyvinylidene fluoride (PVDF) membranes at 4 °C (300 mA for 150 min). The PVDF membranes were blocked in 5% skim milk in TBST for 1 h and incubated overnight with C1QTNF6 (ab115455, 1:500, Abcam, Cambridge, MA, USA), ID1 (1:400, ab134163, Abcam), JAK2 (1:400, #3230, Cell Signaling Technology), DDIT3 (1:200, ab11419, Abcam), MK167 (1:300, ab15580, Abcam), or BBC3 (1:1000, #12450, Cell Signaling Technology) at 4 °C. After three washes in TBST, the membranes were incubated with secondary antibody coupled to HRP (1:1000, sc-2005, Santa Cruz) before visualization. β-actin (1:1,000, sc-32233, Santa Cruz) was used as reference.

### Immunohistochemistry

OSCC tissues and normal tissues were immunostained for C1QTNF6 expression using a C1QTNF6 antibody (Invitrogen, USA) at a 1:100 dilution for 1 h according to the supplier’s instructions. The secondary antibody (goat anti-rabbit IgG) was added for 1 h at 37 °C. Images were obtained at the optical facility. Immunohistochemical staining of C1QTNF6 was evaluated semi-quantitatively by percentage of staining cells and staining intensity. The intensity score was defined as follows: 0, no appreciable staining; 1, weak intensity; 2, moderate intensity; 3, strong intensity; 4, very strong intensity. The staining percentage score was based on the proportion of positively stained cells (0–100%).

### Celigo

Lentivirus-transfected cells were seeded into 96-well plates. Cell density was 2,000 cells/well in incubator with 37˚C and 5% CO2. Starting from the second day after laying the board, Celigo will test the reading board once a day for 3–5 consecutive days. By adjusting the input parameters of Analysis Settings, the number of cells with green fluorescence in each scanning orifice was accurately calculated. The data were statistically plotted and the cell proliferation curve for 5 days was plotted. Normalization process of celigo experiment is as follows: the cell count values of each group at each time point were calculated and compared with those of the group on the first day, so as to obtain the proliferation multiple of the cells in this group at each time point. According to the proliferation multiple and points in time, the growth curve based on cell proliferation multiple was drawn.

### Cell cycle analysis by flow cytometry

Lentivirus-transfected cells were seeded in 6-cm dishes. When the cells achieved approximately 80% confluence, they were trypsinized, washed twice in PBS, and fixed with pre-chilled 70% ethanol for at least 1 h at 4 °C. Then the cells were washed with PBS and stained with PI mixture (40 × PI stock (2 mg/ml), 100 × RNase stock (10 mg/ml) and 1 × PBS buffer at a dilution of 25:10:1,000) for 45 min at 37 °C. The cell cycle results were measured on a Guava easyCyte HT (Millipore, USA), and all experiments were performed in triplicate.

### MTT assay

MTT assay was used to measure the cell viability. Briefly, Lentivirus-transfected cells (2000 cells/well) were seeded in five 96-well plates for test last 5 days. MTT (Genview,USA) solution (20 μl) was added to a final concentration of 5 mg/ml, and the cells were incubated for 4 h. The medium was then removed, and 150 μl of dimethylsulfoxide (DMSO) (Shiyi, Shanghai, China) was added and incubated for 30 min at room temperature. The absorbance of the samples was measured at 490 nm using a microplatereader (Tecan Group Ltd., Mnnedorf, Switzerland).

### Annexin V apoptosis assay

Cell apoptosis analysis was measured using the eBioscience™ Annexin V Apoptosis Detection Kit APC according to the manufacturer’s instructions. Briefly, Lentivirus-transfected cells were trypsinized, washed twice in pre-chilled D-hank’s, and resuspended in binding buffer. Then 5 μl of annexin V-APC was added to 100 μl of cell suspension and incubated at room temperature in the dark for 15 min. Samples were analyzed on a Guava easyCyte HT (Millipore, USA).

### In vivo* tumorigenicity*

Four-week-old female BALB/c nude mice were purchased from the Shanghai Institute for Biological Sciences (Shanghai, China). Twenty BALB/c nude mice were randomly divided into two groups (n = 10/group). CaL-27 cells were transfected with either C1QTNF6-shRNA or Ctrl-shRNA lentiviral vector labeled with luciferase. Lentivirus-transfected cells (1 × 10^7^) were inoculated into the right side of the axillary of nude mice subcutaneously. The mice were observed every 5 days. The length and width of tumors were measured using calipers, and the volume of tumors was calculated using the equation (L × W^2^)/2. On day 35, the animals were sacrificed, and the tumors were removed and weighed. Luciferase activity in tumor tissue was detected by IVIS LuminaXR system (Perkin Elmer, Norwalk, Connecticut, USA).

### Pathway analysis (IPA)

A signal histogram was made to show the signal intensity distribution of all chip probes, and the average Z-score value of all samples in the same signal intensity interval was less than 2. Relative Signal Box Plot analysis also showed that the Z-score value of the medians was less than 2. Correlation Analysis was performed according to the Pearson correlation coefficient distribution chart, which indicated that the correlation coefficients within both groups were greater than 0.99. Principal component analysis was further conducted. All the above analysis confirmed that the data displayed reliability, repeatability, and significant differences between groups, thus meeting our criteria for continued analysis. The lowest 20% of the signal intensity of the probe set was filtered out as background noise. Next, we used the coefficient of variation method to calculate the variability of the same probe in the same sample and filtered out probes with a coefficient of variation greater than 25% in both groups. Finally, our dataset included 39,287 probes.

We used a linear model based on the empirical Bayes distribution to calculate the significant difference level P-value and used the Benjamini–Hochberg method to correct the significant difference level. The screening criteria for significantly different genes were |Fold Change|> 1.5 and FDR < 0.05. Hierarchical Clustering was performed, with the clustering algorithm classifying the samples and variables in two dimensions.

Next, we used the Ingenuity Pathways Analysis (IPA, Ingenuity systems, Inc., Redwood City, CA, www.ingenuity.com) tool to examine biological functions and disease correlations as well as functional relationships between genes and gene networks.

### Statistical analysis

All Statistical analyses were performed using GraphPad Prism software (La Jolla, CA, USA). Data were expressed as the mean ± standard deviation (SD) and the statistical significance between two groups was assessed using *T*-tests. P < 0.05 was considered statistically significant.

## Results

### C1QTNF6 is highly expressed in OSCC tissue and cell lines

**Fig. 1:**
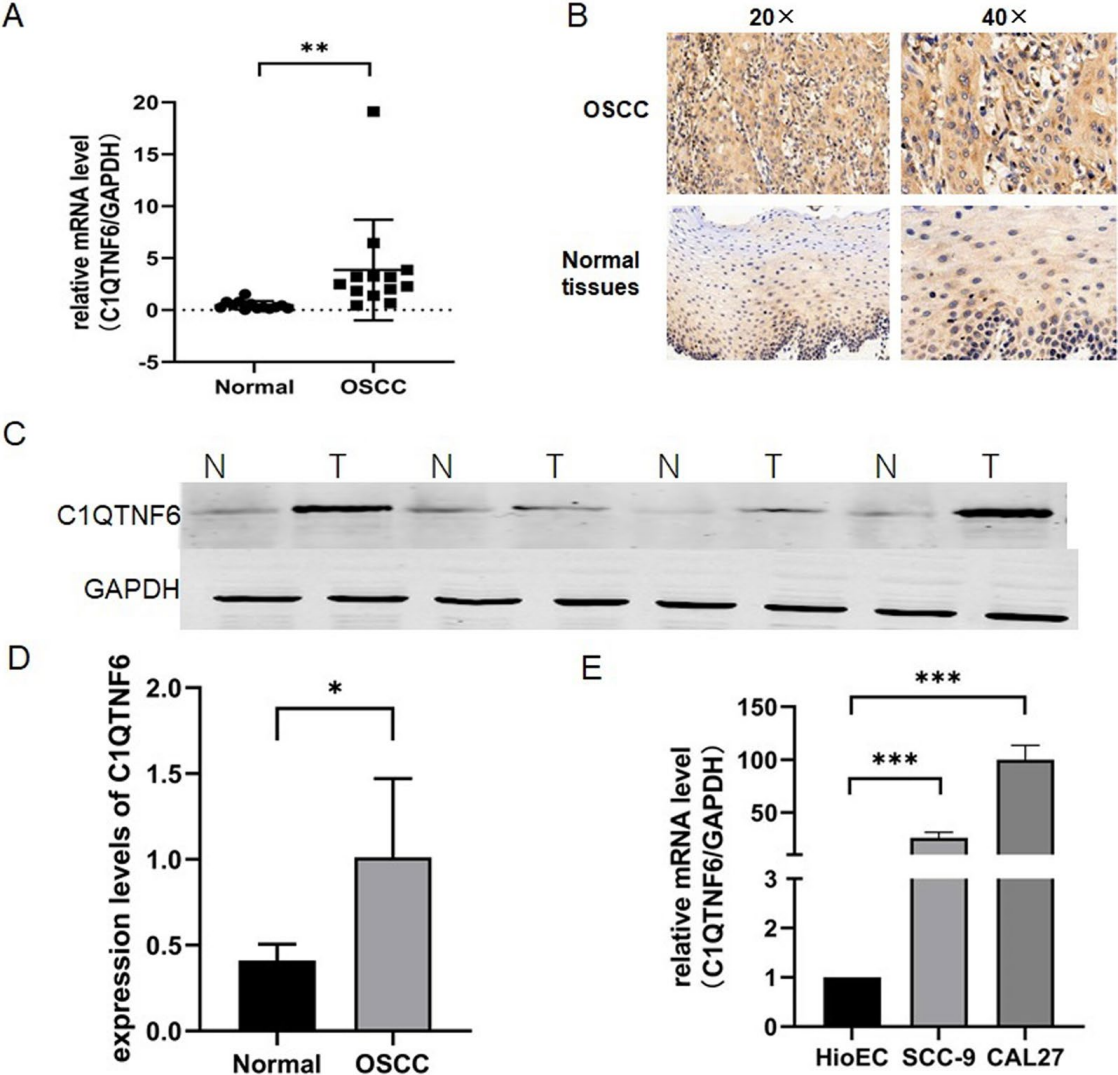
C1QTNF6 expression is upragulated in OSCC. **A** The mRNA expression levels of C1QTNF6 in 13 pairs of human OSCC tissues and the paired normal tissues were detected by qRT-PCR. **B** Detection of C1QTNF6 expression in human OSCC and paracancerous tissues by immunohistochemical staining. **C **Detection of C1QTNF6 protein expression in OSCC and paracancerous tissues by western blot. GAPDH was used as a internal control. **D **The protein level of C1QTNF6 in OSCC was significantly higher than that in paracancerous tissues **E** The mRNA expression levels of C1QTNF6 in HioEC, Cal-27 and SCC-9 cells were detected by qRT-PCR. All statistics are mean ± SD.*P < 0.05,**P < 0.01, ***P < 0.001

To determine whether C1QTNF6 may have a role in OSCC, we downloaded and analyzed data from 40 paired cancer and normal tissues from The Cancer Genome Atlas (TCGA). The results suggest that C1QTNF6 tends to be more highly expressed in OSCC, indicating that it could potentially be a tumor-associated regulator involved in the carcinogenesis of OSCC. As additional verification, we collected 13 paired OSCC and normal tissues and observed the levels of C1QTNF6 by qRT-PCR. Compared with adjacent normal tissues, the expression of C1QTNF6 was higher in the OSCC tissues (Fig. 1A). We assessed C1QTNF6 expression in OSCC tissue and matched adjacent normal tissues using immunohistochemical staining, the results of which were consistent with qRT-PCR (Fig. 1B). Further detection of the expression of C1QTNF6 in OSCC and paracancerous tissues by western blot also proved that the expression of C1QTNF6 in OSCC was significantly higher than that in normal tissues (Fig. 1C, D). We selected HioEC as the oral normal epithelial cell line, Cal-27 and SCC-9 as the human OSCC cell lines to detect C1QTNF6 mRNA levels by qRT-PCR. It was found that the expression of C1QTNF6 in Cal-27 and SCC-9 lines were significantly higher than that in HioEC (Fig. 1E). These results indicate that C1QTNF6 is expressed in OSCC and that its expression may correlate with disease status.

### Cell proliferation is inhibited by C1QTNF6 knockdown in human OSCC cell lines

**Fig. 2:**
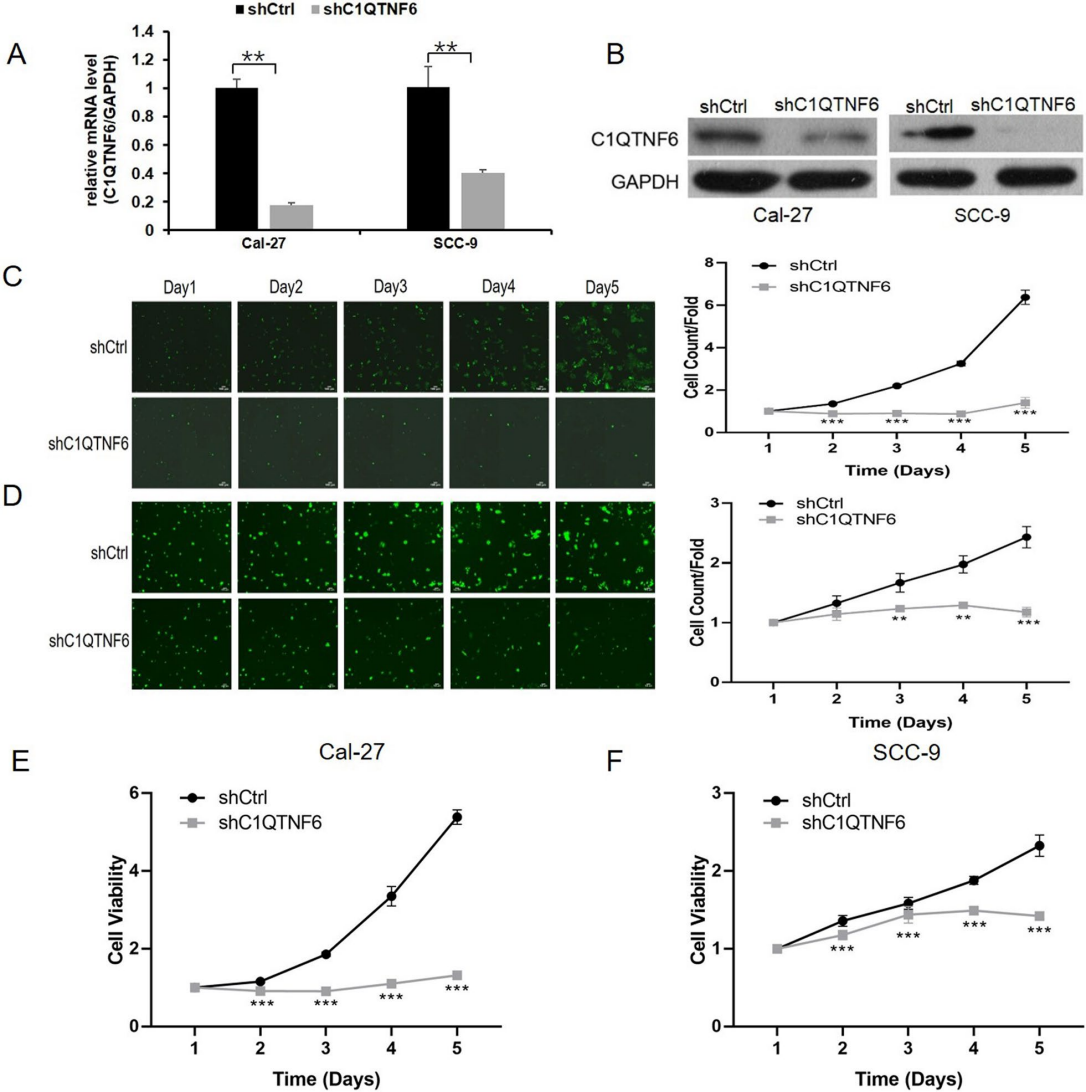
Knockdown of C1QTNF6 inhibits cell proliferation in OSCC cell lines. mRNA (**A**) and protein (**B**) levels of C1QTNF6 in CAL-27 and SCC-9 cells transfected with LV-Ctrl-shRNA or LV-C1QTNF6-shRNA. GAPDH was used as internal control. Cell growth was measured by Celigo assay over five days. Fluorescent images are shown for Cal-27 cells (**C**) and SCC-9 cells (**D**) (magnification, × 200,scale bar,100 μm) transfected with LV-Ctrl-shRNA (shCtrl) or LV-C1QTNF6-shRNA (shC1QTNF6). The cell growth curves (C&D right panels) show the cell number fold from days 1 to 5. Knockdown of C1QTNF6 inhibits proliferation of Cal-27 (**E**) and SCC-9 cells (**F**) as assessed using MTT assay.All statistics are represented as mean ± SD.**P < 0.01, ***P < 0.001

To explore the biological role of C1QTNF6 in OSCC tumorigenesis, we constructed a lentiviral-based shRNA to knockdown C1QTNF6 in Cal-27 and SCC-9 cells. More than 90% of siRNA-transfected cells expressed GFP under a fluorescent microscope at 48 h after lentivirus infection, which suggests a high infection efficiency (data not shown). The efficiency of the C1QTNF6 knockdown was confirmed by qRT-PCR and western blotting. C1QTNF6 mRNA levels were significantly reduced in both cell lines after C1QTNF6-shRNA transfection, with approximately 60–80% knockdown efficiency (Fig. 2A). Furthermore, western blot analysis demonstrated dramatically decreased C1QTNF6 protein expression in C1QTNF6-shRNA transfected cells compared to Ctrl-shRNA transfected cells (Fig. 2B).

Sustained proliferation is a hallmark of cancer cells. To determine the effect of the C1QTNF6 gene on the cell proliferation of OSCC cell lines, we performed two assays to evaluate cell proliferation. For the Celigo assay, viable cells were counted every day for five consecutive days after infection with LV-C1QTNF6-shRNA or LV-Ctrl-shRNA. Silencing of C1QTNF6 lowered the cell growth curve from day 3 to day 5 in Cal-27 (Fig. 2C) and SCC-9 cells (Fig. 2D). Furthermore, in both cases, the inhibition of cell growth by C1QTNF6 silencing was more pronounced over time. For the MTT assay, the optical density at 490 nm increased by 3.3-fold in Cal-27 cells after treatment with Ctrl-shRNA and 1.1-fold after treatment with C1QTNF6-shRNA at 4 days after transfection (Fig. 2E). Similar results were obtained in SCC-9 cells (Fig. 2F). Together, the data indicate that the knockdown of C1QTNF6 significantly inhibits cell proliferation in human OSCC cell lines.

### C1QTNF6 knockdown induces cell cycle arrest and apoptosis of human OSCC cell lines

**Fig. 3:**
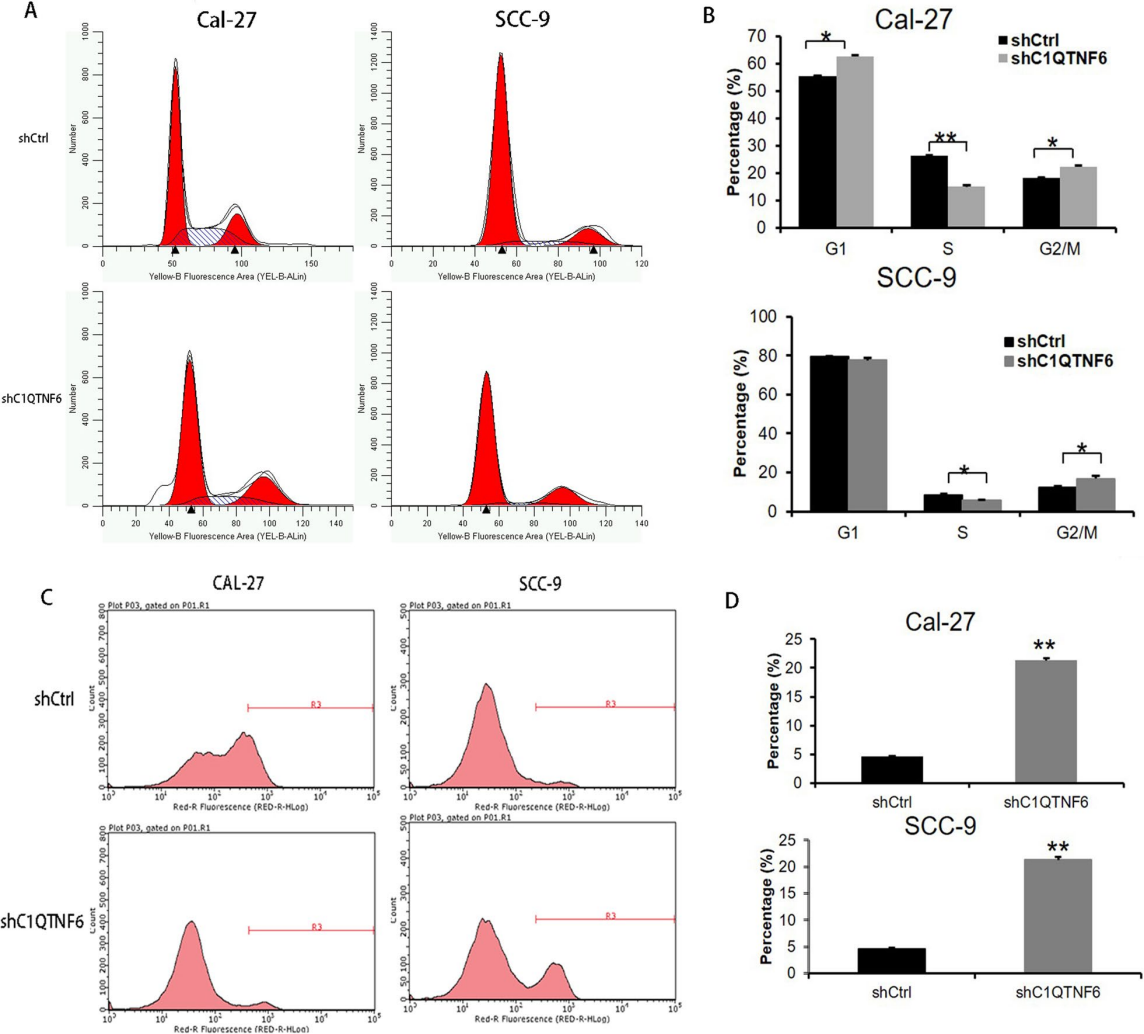
Knockdown of C1QTNF6 induces G2/M phase arrest and cell apoptosis in OSCC cell lines. Cell cycle distribution of Ctrl-shRNA and C1QTNF6-shRNA transfected cells was examined using PI staining and flow cytometry. **A** The representative cell cycle distribution of PI-stained cells. **B** The average percentage of cells in the G0/G1, S, and G2/M phases is shown. **C** Representative flow cytometry images of Annexin V-APC-stained cells are shown. **D** The percentages of apoptotic cells are shown. All statistics are represented as mean ± SD. *P < 0.05, **P < 0.01

To determine whether the effect of C1QTNF6 on the proliferation of OSCC cell lines was accounted for by its ability to promote cell cycle stability or inhibit apoptosis, we used PI staining to measure the cell cycle distribution and Annexin-V staining to assess apoptosis in Cal-27 and SCC-9 cells modified by Ctrl-shRNA or C1QTNF6-shRNA. As shown in Fig. 3A, B, Ctrl-shRNA transfected Cal-27 cells had a cell cycle distribution of 55.47% G0/G1, 26.28% S, and 18.25% G2/M; knockdown of C1QTNF6 significantly reduced the fraction of cells in the S phase and significantly increased the fraction in the G2/M phase, with 62.77% G0/G1, 14.94%S, and 22.92% G2/M. These data demonstrate that the cell cycle progression through the G2/M phase was hindered in Cal-27 cells after C1QTNF6 silencing. SCC-9 cells transduced with Ctrl-shRNA had the following cell cycle distribution: G0/G1 79.35%, S 8.53%, G2/M 12.12%; C1QTNF6-shRNA knockdown significantly reduced the fraction of cells in the S phase, and significantly increased the fraction in the G2/M phase, with the following cell cycle distribution: G0/G1 77.54%, S 5.55%, G2/M 16.91%. Furthermore, the apoptosis of Cal-27 cells and SCC-9 cells was significantly increased by C1QTNF6 knockdown (Fig. 3C, D). These results suggest that C1QTNF6 silencing impedes cell cycle progression and promotes apoptosis in OSCC cell lines.

### *C1QTNF6 promotes human OSCC cell growth *in vivo

**Fig. 4:**
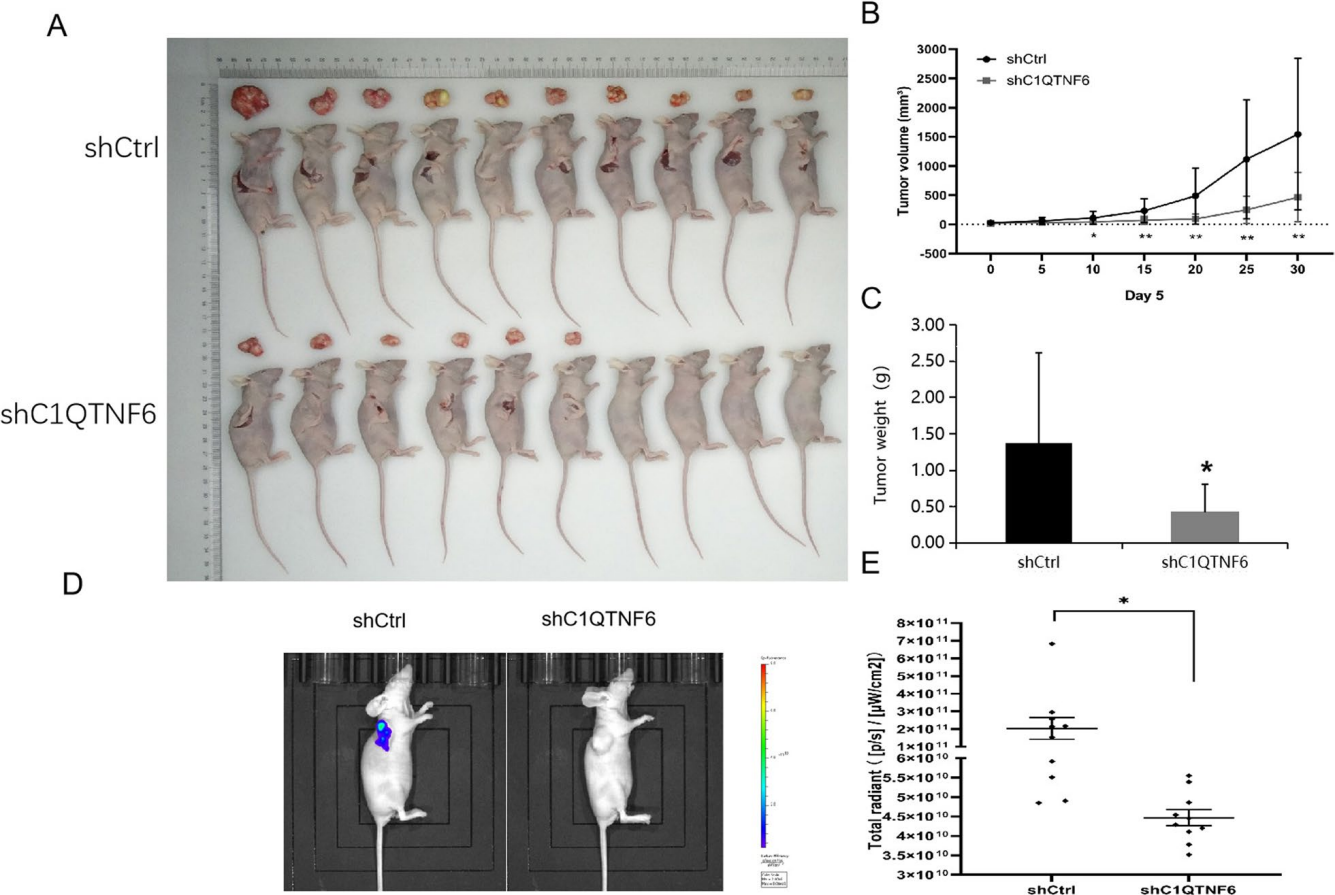
Knockdown of C1QTNF6 suppresses tumor growth in vivo.** A** Xenograft models in nu/nu mice were generated using Cal-27 cells transfected with Ctrl-shRNA (n = 10) or C1QTNF6-shRNA (n = 10). **B** Tumor volume was measured every 5 days for 35 days. **C** The average weights of excised tumors are shown. Data represent the mean ± SD (n = 10). *P < 0.05, **P < 0.01. **D ** After injection, luminescence was detected in 10 nude mice in each group and representative images of each group were displayed. **E** Compared with the Ctrl-shRNA group, the fluorescence expression of the C1QTNF6-shRNA group decreased*P < 0.05

To extend the in vitro observations, we investigated the effects of C1QTNF6 knockdown on tumor growth in vivo. Cal-27 cells that were transfected with either C1QTNF6-shRNA or Ctrl-shRNA lentivirus were injected into nude mice, and the tumor growth was monitored. Tumors formed by C1QTNF6-silenced Cal-27 cells were much smaller during the experimental period than the control tumor (Fig. 4A, B). The total amount of fluorescence expression also reflects the same result (Fig. 4D, E). Furthermore, the excised Ctrl-shRNA tumors weighed on an average of 1300 mg whereas C1QTNF6-shRNA tumors averaged 400 mg (Fig. 4C). Collectively, these results emphasize the in vivo promoting role of C1QTNF6 in cancer progression.

### C1QTNF6 knockdown may inhibit the tumorigenesis of OSCC through activation of the Acute Phase Response signaling pathway and targeting of ID1, BBC3 and DDIT3

**Fig. 5:**
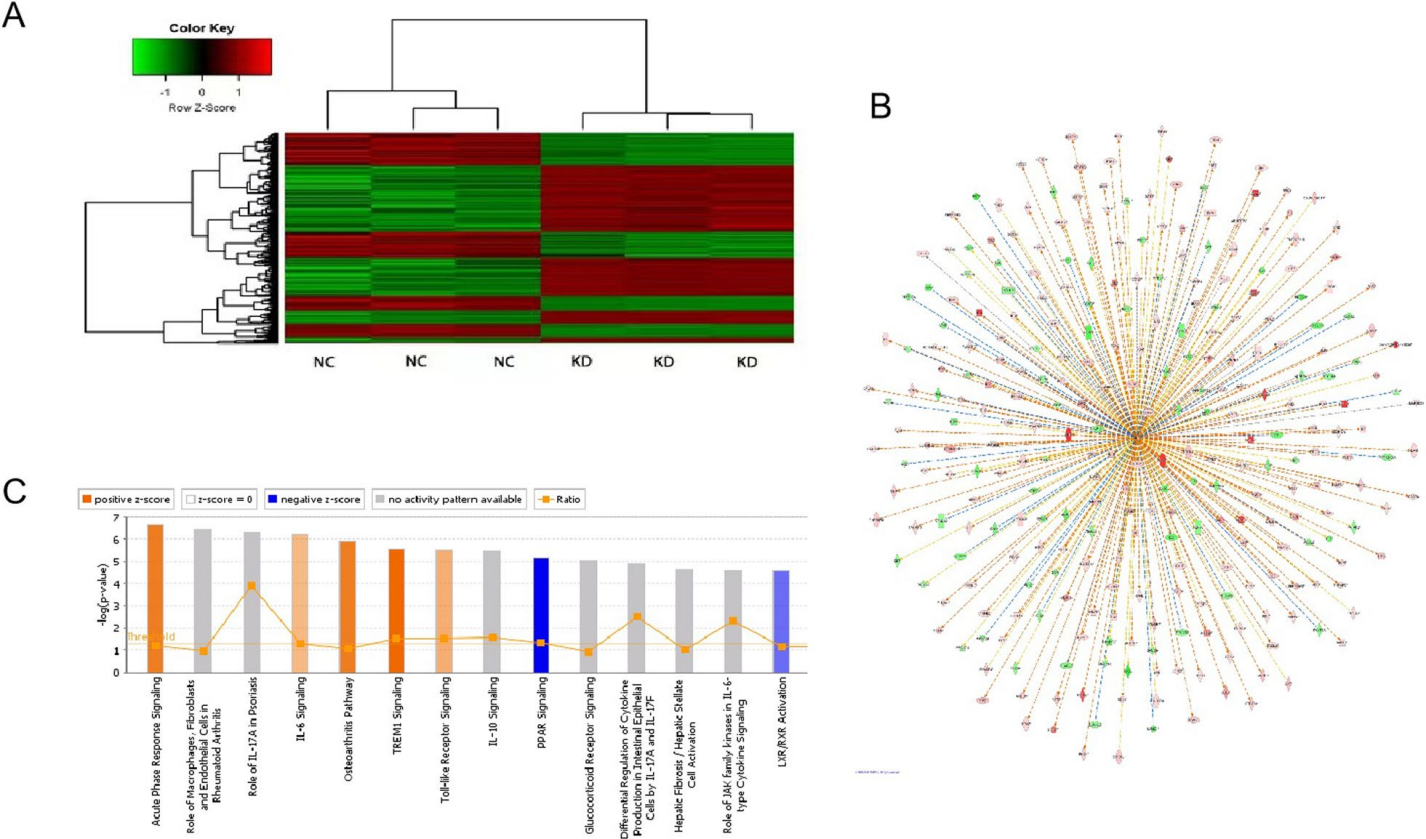
Knockdown of C1QTNF6 activates the Acute Phase Response signaling pathway and targets TNF.** A** Hierarchical clustering analysis heat map of differentially expressed genes. Red indicates upregulation, and green indicates downregulation; black indicates no obvious difference. **B** The TNF network showed the relationships of predicted upstream regulatory factor, TNF and down-stream molecules in the data set. **C** Signaling pathways enriched among differentially expressed genes. The Y-axis represents the − log (10) P value for enrichment, with the threshold drawn at P = 0.05.

**Fig. 6:**
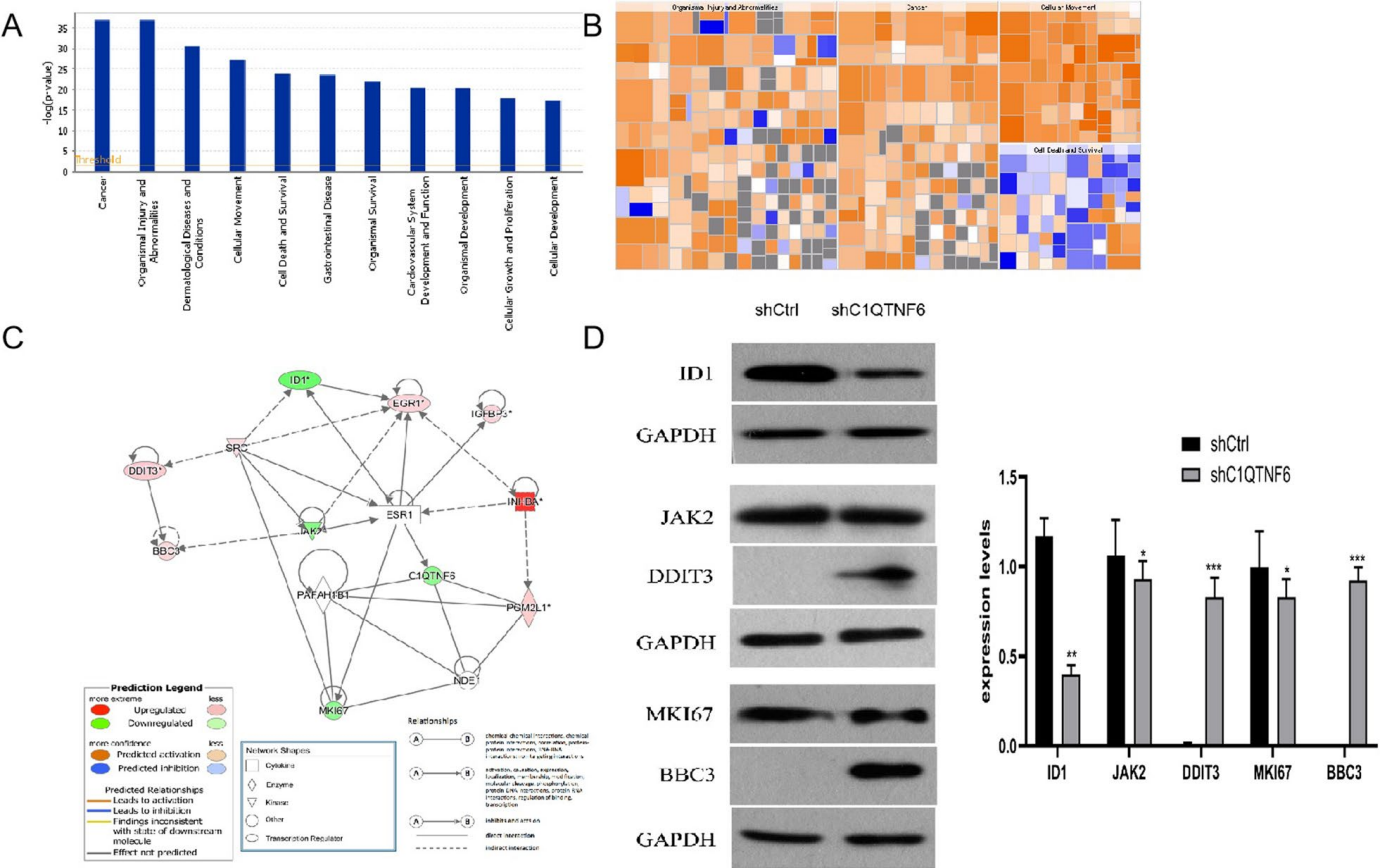
Gene expression profiling revealed the relationship between the expression of C1QTNF6 and ID1, BBC3, DDIT3.** A** The enrichment of genes with significantly different expression in diseases and functions. The abscissa is the disease or the function name, and the ordinate is the significance level of − log (10) value for enrichment. **B** Disease and Function Heat Map illustrates the relationships between the differentially expressed genes and functions and diseases. **C** Interaction network of several molecules closely connected to C1QTNF6, as determined by IPA. **D** Western blot assay showing the expression of ID1, JAK2, DDIT3, MKI67, and BBC3 transfected with Ctrl-shRNA or C1QTNF6-shRNA. Data is represented as mean ± SD, *P < 0.05

The above results suggest that C1QTNF6 plays a critical role in the development of OSCC. To elucidate the molecular mechanisms by which C1QTNF6 promotes malignancy of OSCC cells, we performed microarray analysis to compare gene expression between cells infected with Ctrl-shRNA and C1QTNF6-shRNA lentivirus. The expression of 1628 genes showed significant differential expression (P < 0.05 and |Fold Change|> 1.5), including 943 upregulated genes and 685 downregulated genes. The heat map shows hierarchical clustering analysis of differentially expressed genes, which revealed that OSCC cells from three samples shared similar gene expression patterns after C1QTNF6 knockdown (Fig. 5A). Figure 5B shows the classical signal pathway, demonstrating the clustering status of differentially expressed genes in the classical signal pathway. In this study, the Acute Phase Response signaling pathway was significantly activated following C1QTNF6 silencing. IPA uses the ACTIVATION Z-score algorithm to predict the activation or suppression of upstream regulators and reduce the significant prediction due to random data. In this project, TNF was predicted to be strongly activated. The upstream regulatory subnetwork diagram shows the interactions between TNF and their directly related downstream molecules that coexist in the dataset (Fig. 5C). Disease and function enrichment analysis statistics figure shows the cluster status of the differentially expressed genes in disease and function categories (Fig. 6A). The heat map of disease and function illustrates the relationship between activation and inhibition of disease and function caused by the change of differential gene expression level. As shown in Fig. 6B, the functions that are strongly activated included invasion of cells and invasion of tumor cell lines whereas the functions that were significantly curbed include the organismal death, morbidity or mortality.

Functional connections among C1QTNF6 and several closely related molecules were built based on the existing findings (Fig. 6C). Furthermore, western blot analysis verified the differential expression of several molecules in the above network, including ID1, BBC3, and DDIT3, all of which are known target genes of TNF (Fig. 6D).

## Discussion

C1QTNF6 is a crucial molecular mediator of inflammation, fibrosis, and metabolism [14], and it has also been implied in the involvement of tumor development. The role of C1QTNF6 in tumor cell survival and cell cycle has been investigated in cancers other than OSCC [18, 20]. However, investigations focusing on the relationship between C1QTNF6 and OSCC development remain limited. In the present study, we reported C1QTNF6 is highly more expression in OSCC tissues. In addition, C1QTNF6 knockdown delayed the proliferation of OSCC cells and induced cell apoptosis in vitro. Consistent with our observation, the expression of C1QTNF6 in human gastric carcinoma tissues has been shown to be higher than that in normal gastric tissue, and C1QTNF6 silencing in gastric carcinoma cells decreased the growth and resulted in G2‑M cell cycle arrest [18]. Furthermore, C1QTNF6-related signaling pathways activated in clear cell renal cell carcinoma have been shown to be mainly enriched in DNA replication, Cell Cycle, EMT, and angiogenesis signaling pathways [17]. In line with these observations, we found that C1QTNF6 knockdown increased the percentage of cells at the G2-M phase in human OSCC cell lines. To extend the in vitro observations, we investigated the effect of C1QTNF6 knockdown on tumor growth in vivo and observed that C1QTNF6 silencing suppressed tumor growth in mice. This result further emphasizes the promoting role of C1QTNF6 in OSCC progression.

To date, the molecular mechanisms underlying the function of C1QTNF6 in OSCC remain unclear. To elucidate the molecular mechanisms by which C1QTNF6 promotes malignancy of OSCC, microarray analysis was performed. Hundreds of genes showed significantly different expression between C1QTNF6-shRNA and Ctrl-shRNA transfected cells. According to Ingenuity Pathway Analysis (IPA), the Acute Phase Response was the top-activated signaling pathway following C1QTNF6 silencing. This signaling pathway is an important component of anticancer responses among multicellular organisms [21]. The acute phase response is a nonspecific physiological and biochemical reaction to tissue damage, infection, inflammation, and neoplasia, during which the synthesis of several plasma proteins is increased (positive acute-phase proteins) or decreased (negative acute-phase proteins) [22]. The acute phase response is not diagnostic for any particular disease but occurs as a response to several pathological conditions and diseases, including bacterial infections, sepsis, surgery, trauma, myocardial infarction, inflammatory diseases, and cancer [23]. Other pathways that showed altered activation status after C1QTNF6 knockdown, including TREM1 signaling, IL-6 signaling, and Toll-like Receptor signaling, can induce tumor cell apoptosis or activate anti-tumor immune responses, suggesting that they might be involved in the development of variant cancers [24–26]. Furthermore, Western blot assay showed that ID1 was significantly down-regulated by C1QTNF6 knockdown. In our previous study, we demonstrated that the over-expression of ID1 effectively promotes the carcinogenesis of OSCC [12, 13]. ID1 is an inhibitor of DNA binding (Id) proteins [27]. During development, Id proteins play a key role in the regulation of cell-cycle progression and cell differentiation by modulating different cell-cycle regulators both by direct and indirect mechanisms [28]. ID1 enhances cell proliferation, colony formation, and tumor growth by regulating the cell cycle in lung cancer [29]. Based on these studies, we speculate that the reduced expression of ID1 after C1QTNF6 knockdown further leads to cell cycle arrest in OSCC.

BH3-only protein BBC3 (BCL-2-binding component 3) belongs to the Bcl-2 family and is a strongly proapoptotic gene that is subject to transcriptional regulation by multiple cell death-signaling pathways [30]. It has been shown that BBC3 plays a role in p53-induced apoptotic pathways and can also function independently of p53. DDIT3, also known as C/EBP homologous protein (CHOP) or growth arrest and DNA damage-inducible gene 153 (GADD153), is a member of the CCAAT/enhancer-binding protein (C/EBP) family [31]^.^ DDIT3 (DNA-damage inducible transcript 3) promotes ovarian cell apoptosis in mice via ER stress activation, and knockdown of DDIT3 suppresses cell apoptosis [32]. DDIT3 is an important transcription factor of apoptosis under ER stress, and the DDIT3-mediated apoptosis pathway is one of the most dominant pathways in the apoptosis process [33]. Over-expression of CHOP has been reported to lead to cell cycle arrest and/or apoptosis, and CHOP has been shown to regulate numerous pro- and apoptotic genes as a transcriptional factor [34]. High levels of BBC3, DDIT3, and obvious cell apoptosis after C1QTNF6 knockdown were observed in this study. Thus, knockdown of C1QTNF6 may induce endoplasmic reticulum stress and promote upregulation of BBC3 and DDIT3, which could explain the increased apoptosis of CIQTNF6-silenced OSCC cell lines in vitro. Therefore, our microarray analysis results fit well with the known effects of signaling pathways and genes on tumorigenesis, suggesting that the promotion of tumor development by C1QTNF6 might be mediated through regulation of Acute Phase Response signaling and ID1, BBC3, and DDIT3 gene expression (Additional files 1, 2).

## Conclusions

There is a balance between the proliferation and apoptosis of tumor cells, and the regulation of this balance determines the development and progression of the tumor. Based on our findings, we speculate that C1QTNF6 knockdown leads to complex changes in OSCC, which produces an anti-cancer effect through acute phase response both in vivo and in vitro. Microarray analysis suggests that changes in the expression of target genes, including ID1, BBC3, and DDIT3, could cause cell cycle arrest and promote apoptosis of OSCC. Our study provides a possible molecular mechanism underlying C1QTNF6-mediated promotion of OSCC tumorigenesis, which needs to be further studied, especially cell cycle factors, apoptosis proteins, and ER stress. Our findings not only provide a novel target gene for OSCC therapy but also add new evidence supporting the relationship between C1QTNF6 and tumorigenesis (Additional files 3, 4).

## Supplementary Information

**Additional file 1: Figure S1.**. The effects of C1QTNF6 knockdown on protein expression in xenograft tumor tissues according to westernblot. GAPDH was used as a loading control. All results were reproducible in three independent experiments. *P<0.05, **P<0.01.

**Additional file 2: Figure S2.**. Xenograft models in nu/nu mice were generated using Cal-27 cells transfected with Ctrl-shRNA or C1QTNF6-shRNA. luminescence was detected , and representative images of each group were displayed.

**Additional file 3: Figure S3.**. Silencing C1QTNF6 induced cleaved caspase3 and parp. Silencing C1QTNF6 increased the cleaved caspase3 (A) and parp (B) related with apotosis in SCC-9 cells. *P<0.05, **P<0.01, ***P<0.001.

**Additional file 4**. The total amount of fluorescence expression of the C1QTNF6-shRNA group and Ctrl-shRNA group.

## Data Availability

The datasets used and analyzed in the current study are available from the corresponding author in response to reasonable requests.
