# Supplementary figures and images for: C1QTNF6 promotes oral squamous cell carcinoma by enhancing proliferation and inhibiting apoptosis
Source: Cancer Cell Int. 2021 Dec 14;21:666. doi: 10.1186/s12935-021-02377-x (PMC8670214; doi:10.1186/s12935-021-02377-x)

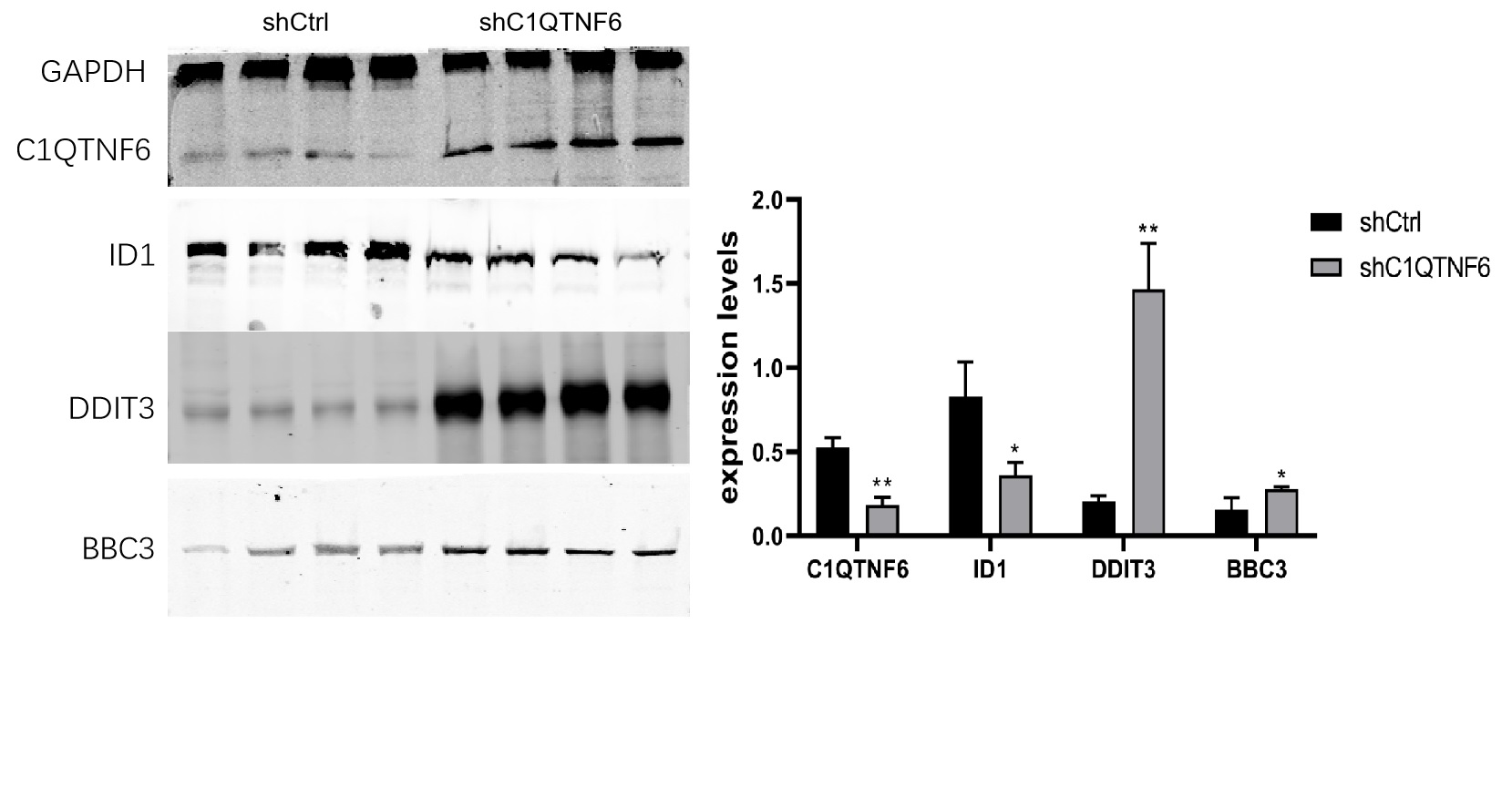

Supplement: Supplementary file 1 — Additional file 1: Figure S1.. The effects of C1QTNF6 knockdown on protein expression in xenograft tumor tissues according to westernblot. GAPDH was used as a loading control. All results were reproducible in three independent experiments. *P<0.05, **P<0.01. [file 12935_2021_2377_MOESM1_ESM.jpg]

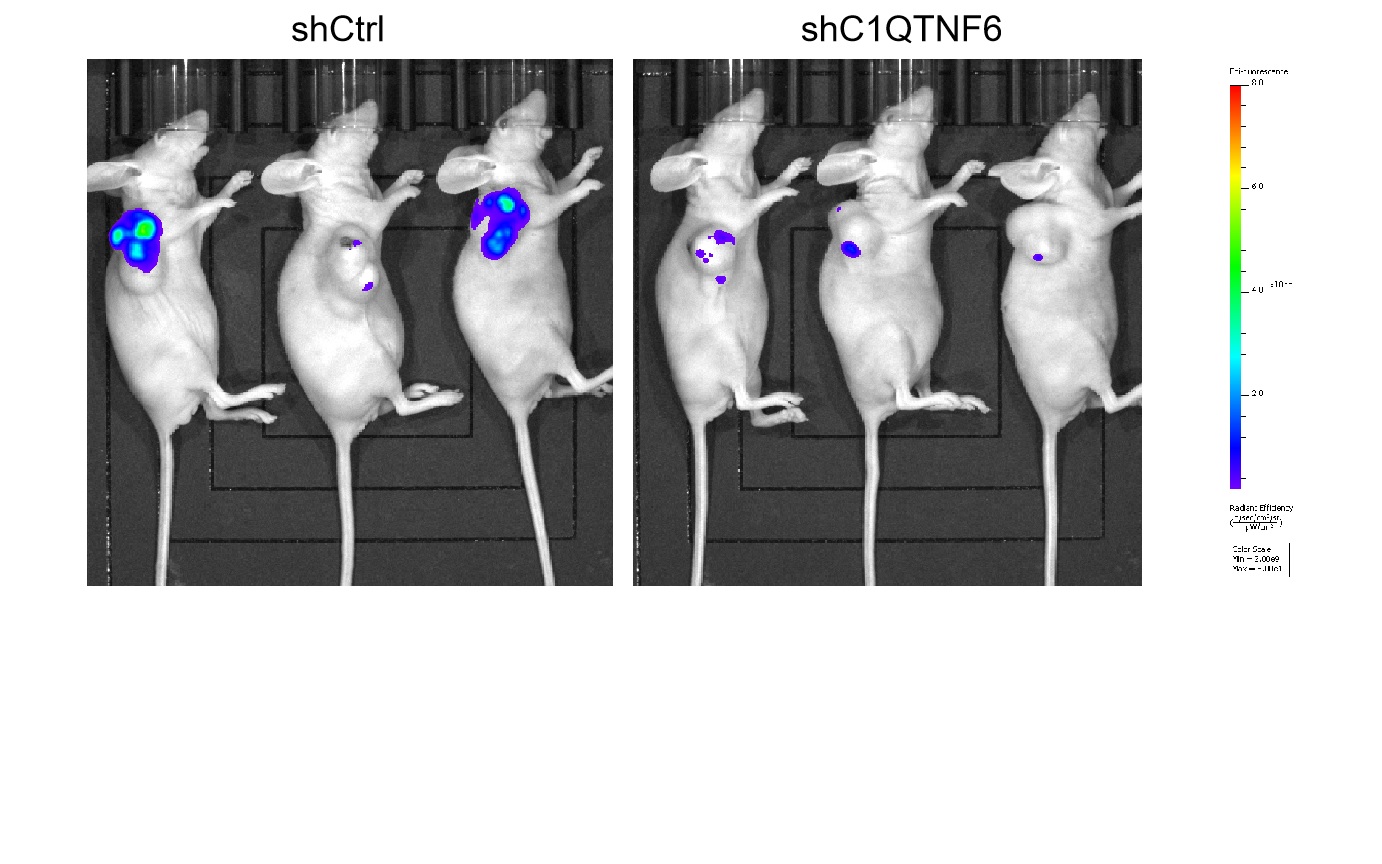

Supplement: Supplementary file 2 — Additional file 2: Figure S2.. Xenograft models in nu/nu mice were generated using Cal-27 cells transfected with Ctrl-shRNA or C1QTNF6-shRNA. luminescence was detected , and representative images of each group were displayed. [file 12935_2021_2377_MOESM2_ESM.jpg]

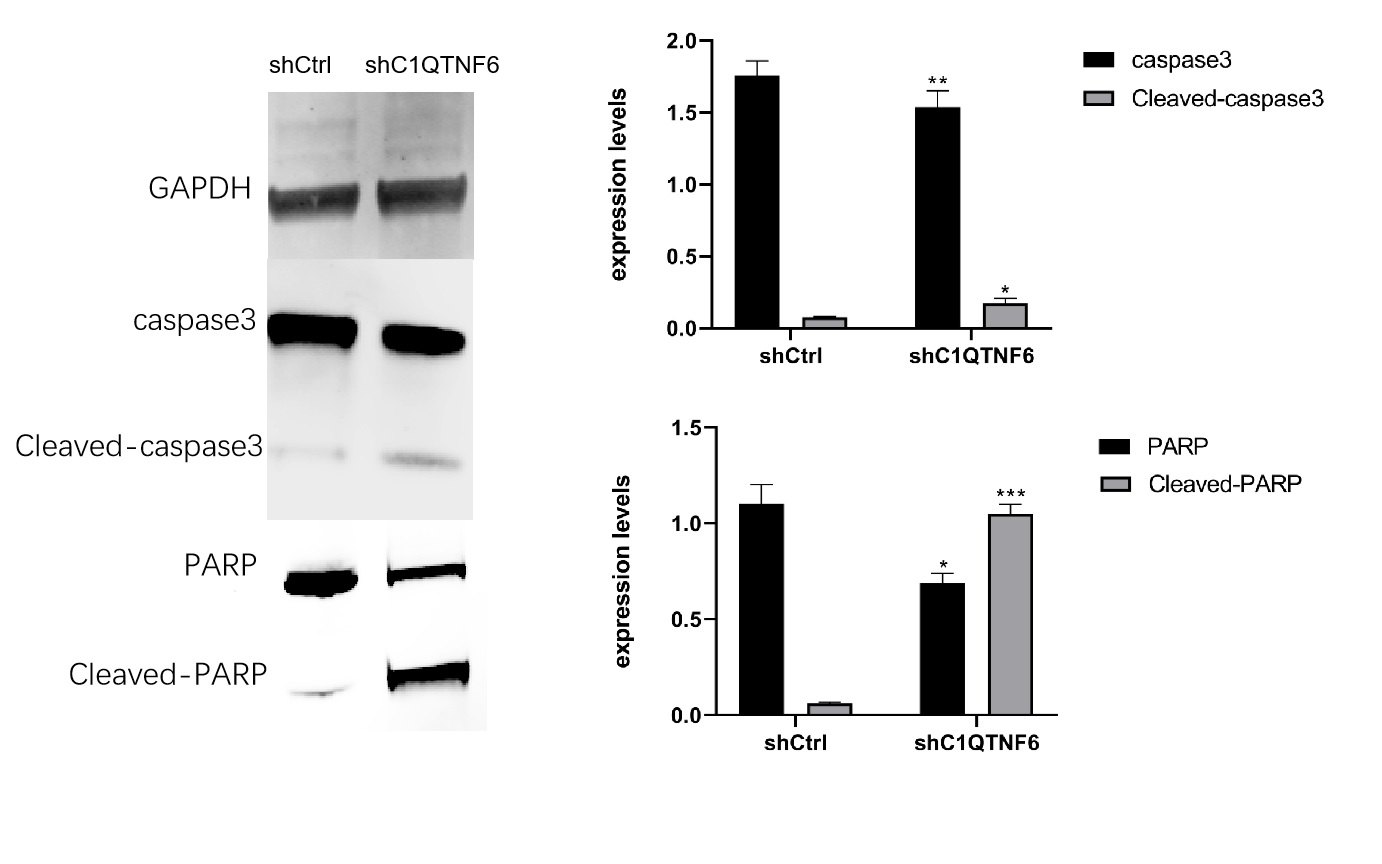

Supplement: Supplementary file 3 — Additional file 3: Figure S3.. Silencing C1QTNF6 induced cleaved caspase3 and parp. Silencing C1QTNF6 increased the cleaved caspase3 (A) and parp (B) related with apotosis in SCC-9 cells. *P<0.05, **P<0.01, ***P<0.001. [file 12935_2021_2377_MOESM3_ESM.jpg]
